# A cross-sectional seroepidemiological survey of typhoid fever in Fiji

**DOI:** 10.1371/journal.pntd.0005786

**Published:** 2017-07-20

**Authors:** Conall H. Watson, Stephen Baker, Colleen L. Lau, Kitione Rawalai, Mere Taufa, Jerimaia Coriakula, Nga Tran Vu Thieu, Tan Trinh Van, Dung Tran Thi Ngoc, Niel Hens, John H. Lowry, Ruklanthi de Alwis, Jorge Cano, Kylie Jenkins, E. Kim Mulholland, Eric J. Nilles, Mike Kama, W. John Edmunds

**Affiliations:** 1 Department of Infectious Disease Epidemiology, London School of Hygiene & Tropical Medicine, London, United Kingdom; 2 The Hospital for Tropical Diseases, Wellcome Trust Major Overseas Programme, Oxford University Clinical Research Unit, Ho Chi Minh City, Vietnam; 3 Centre for Tropical Medicine and Global Health, Nuffield Department of Clinical Medicine, Oxford University, Oxford, United Kingdom; 4 Department of Global Health, Research School of Population Health, Australian National University, Canberra, Australia; 5 Project Heaven, Suva, Fiji; 6 Fiji Centre for Communicable Disease Control, Ministry of Health and Medical Services, Suva, Fiji; 7 Pacific Research Center for the Prevention of Obesity and Non-Communicable Diseases, Fiji National University, Suva, Fiji; 8 Center for Statistics, I-Biostat, UHasselt, Hasselt, Belgium; 9 Centre for Health Economic Research and Modelling Infectious Diseases, Vaccine and Infectious Disease Institute, University of Antwerp, Antwerp, Belgium; 10 School of Geography, Earth Science and Environment, University of the South Pacific, Suva, Fiji; 11 Department of Disease Control, London School of Hygiene & Tropical Medicine, London, United Kingdom; 12 Fiji Health Sector Support Programme, Suva, Fiji; 13 Telethon Kids Institute, Perth, Western Australia, Australia; 14 Infection and Immunity, Murdoch Childrens Research Institute, Melbourne, Australia; 15 Emerging Disease Surveillance and Response, World Health Organization Western Pacific Region, Suva, Fiji; Emory University, UNITED STATES

## Abstract

Fiji, an upper-middle income state in the Pacific Ocean, has experienced an increase in confirmed case notifications of enteric fever caused by *Salmonella enterica* serovar Typhi (*S*. Typhi). To characterize the epidemiology of typhoid exposure, we conducted a cross-sectional sero-epidemiological survey measuring IgG against the Vi antigen of *S*. Typhi to estimate the effect of age, ethnicity, and other variables on seroprevalence. Epidemiologically relevant cut-off titres were established using a mixed model analysis of data from recovering culture-confirmed typhoid cases. We enrolled and assayed plasma of 1787 participants for anti-Vi IgG; 1,531 of these were resident in mainland areas that had not been previously vaccinated against *S*. Typhi (seropositivity 32.3% (95%CI 28.2 to 36.3%)), 256 were resident on Taveuni island, which had been previously vaccinated (seropositivity 71.5% (95%CI 62.1 to 80.9%)). The seroprevalence on the Fijian mainland is one to two orders of magnitude higher than expected from confirmed case surveillance incidence, suggesting substantial subclinical or otherwise unreported typhoid. We found no significant differences in seropositivity prevalences by ethnicity, which is in contrast to disease surveillance data in which the indigenous iTaukei Fijian population are disproportionately affected. Using multivariable logistic regression, seropositivity was associated with increased age (odds ratio 1.3 (95% CI 1.2 to 1.4) per 10 years), the presence of a pit latrine (OR 1.6, 95%CI 1.1 to 2.3) as opposed to a septic tank or piped sewer, and residence in settlements rather than residential housing or villages (OR 1.6, 95% CI 1.0 to 2.7). Increasing seropositivity with age is suggestive of low-level endemic transmission in Fiji. Improved sanitation where pit latrines are used and addressing potential transmission routes in settlements may reduce exposure to *S*. Typhi. Widespread unreported infection suggests there may be a role for typhoid vaccination in Fiji, in addition to public health management of cases and outbreaks.

## Author summary

Fiji has experienced a decade-long increase in typhoid fever cases, a potentially life-threatening systemic bacterial disease caused by *Salmonella* Typhi. We undertook a representative blood-serum community survey to measure antibodies (IgG) against the Vi antigen of *Salmonella* Typhi using a rigorous survey design. We found one in three residents of mainland, unvaccinated Fiji had detectable antibody against Vi. This was higher than would be expected from confirmed case notifications received by the national surveillance system. Additionally, similar antibody responses were detected in Fijians of all ethnicities, which contrasts to surveillance cases in which indigenous iTaukei Fijians were disproportionately affected. Serology on a Fijian island where a significant proportion of the population has been vaccinated found that three-quarters of residents were seropositive three years after the Vi-polysaccharide typhoid vaccination campaign. Importantly, in mainland participants, seroprevalence increased with age, suggesting long-standing, low-level, endemic transmission. Pit latrines were associated with seropositivity when compared with septic tanks, and settlements compared with residential housing. Very high antibody titres in a small percentage of participants may suggest carriage of *Salmonella* Typhi. The seroprevalence findings suggest eliminating typhoid from Fiji by focussing on cases and outbreaks alone will be challenging. Our results support typhoid vaccination and further development of water, sanitation and hygiene infrastructure in Fiji.

## Introduction

Typhoid fever is a systemic disease resulting from infection by the bacterium *Salmonella enterica* subspecies *enterica* serovar Typhi (*S*. Typhi), a human restricted pathogen transmitted from faeces to water and food or by contact and fomites. [1,2] Infection syndromes range from asymptomatic (including carriage) to severe disease with life-threatening complications, including intestinal perforation, encephalopathy, and haemodynamic shock [3,4]. Many typhoid cases present as non-specific acute febrile illnesses that may be difficult to differentiate from other common tropical infectious diseases such as dengue and leptospirosis. There were an estimated 11.9 million (9.9 to 14.7) cases of typhoid in low and middle income countries in 2010, resulting in 129,000 (75,000 to 208,000) deaths [5].

Fiji is an upper middle income country with an estimated population of 892,000 in 2015 [6] across 100 inhabited Pacific Ocean islands, predominantly residing on Viti Levu and Vanua Levu [7]. Administratively, Viti Levu comprises the Western and Central Divisions, the latter containing the capital Suva. Northern Division comprises of Vanua Levu and Taveuni island, whilst Eastern Division comprises of smaller island groups.

Phylogenetic evidence from genome sequencing suggests that *S*. Typhi has a long history in Fiji though notified blood-culture confirmed cases numbered fewer than 30 annually up to 2004 [8,9]. Annual blood-culture confirmed cases notified through Divisional hospitals to national surveillance have risen [9,10], from 4.4 cases per 100,000 population in 2004 to 45 cases per 100,000 during 2008–2011 [11]. Northern Division saw 121 cases per 100,000 in 2009 vs 28 per 100,000 in the West and 19 per 100,000 in Central [11]. A proportionate increase in clinically diagnosed typhoid was also reported [12].

Notably, >90% of blood-culture confirmed cases are reported amongst indigenous Fijians (iTaukei, approximately 57% of the population), with few cases reported in Fijians of Indian descent (Indo-Fijians, 38% of the population) or Fijians of Asian or European descent[12]. The causes of this disparity are unknown [12]; health seeking behaviours would be expected to lead to higher relative ascertainment in Indo-Fijians than iTaukei [13].

Historically subsistent through agriculture and fishing, more than half the population now reside in urban areas, including informal settlements close to riverbanks and other flood-prone areas with limited access to water and sanitation infrastructure [14].

Typhoid vaccine is not routinely used in Fiji; however, in 2010, following cyclone Tomas, a Vi-polysaccharide (Vi-PS) vaccination campaign was conducted in the highest incidence areas of Fiji. These were predominantly in the Northern Division, with high coverage on Taveuni island and in the Cakaudrove subdivision on Vanua Levu, with targeted geographical vaccination within subdivisions elsewhere [11] reaching 64,000 people, 8% of the national population. Observed disease incidence rates declined in the targeted areas against increasing or stable rates in other areas[11]. Given the ongoing typhoid transmission and the short duration of Vi-PS protection,[15] a 2012 expert meeting was convened by the Ministry of Health, with Australian Aid support, to “develop, prioritise and implement a comprehensive control and prevention strategy” [12]. Knowledge gap analysis identified that a serological survey could inform vaccination policy [12].

Seroepidemiological surveys can help determine population immunity, pathogen exposure and susceptibility, as well as disease- and exposure-related risk factors [16]. Conducted alongside clinical and/or laboratory surveillance, seroepidemiology can help quantify surveillance under- or over-ascertainment, including for enteric diseases [17–20]. Typhoid transmission dynamics are influenced by setting-specific immunity and carriage [21–23]; however, the seroepidemiological methods to attain these are underexploited [17]. This may be in part due to concerns about the sensitivity and specificity of serology for typhoid, which historically has not demonstrated sufficient discriminatory power for individual-level clinical diagnosis [24], (though recent methods may offer promise [25]) as well as concerns about the specificity of assays for carriage detection [26–28] and the existence of multiple immunological pathways to immunity against typhoid fever [29]. Serosurveys utilising assays based on purified, pharmaceutical-grade Vi polysaccharide, the “virulence” factor expressed by *S*. Typhi [27], for detection of anti-Vi IgG antibody may offer a more reliable approach by avoiding cross-reactivity that arises when Vi antigen preparations contain other bacterial antigens [30,31]. Furthermore, high anti-Vi titres may indicate prolonged immune stimulus from chronic carriage [30,32–34]. We undertook a joint typhoid and leptospirosis seroepidemiological survey [35], and present typhoid findings here.

## Methods

### Ethics statement

The study was approved by the Fiji National Research Ethics Review Committee (2013–03) and the London School of Hygiene & Tropical Medicine observational study research ethics committee (6344). All adult participants provided written informed consent. Parents/guardians provided written informed consent on behalf of all child participants (under the age of 18 years old). Written assent was also provided by children aged 12 years and above.

### Study design

To characterize the immunoepidemiology of typhoid infection in Fiji, with the aim of informing effective and efficient control measures, we surveyed three groups of people: group 1) unvaccinated areas of the main Fijian islands; 2) residents of Taveuni island, where a high-coverage vaccination campaign was done in 2010 [11]; and group 3) a cohort of convalescing Fijian culture-confirmed typhoid cases, to enable estimation of a threshold for seropositivity.

#### Sampling methods

Group 1, Mainland: Sixty-four community clusters were randomly selected by multi-stage, cross-sectional sampling on Viti Levu and Vanua Levu and visited for serological sampling and risk factor questionnaire interviews [36–50] from September to December 2013. Vaccinated areas were excluded to avoid confounding of interpretation of serological responses to natural exposure. Eastern Division (population <40,000) [13], was excluded for logistical reasons. Group 2, Taveuni: A vaccination campaign on Taveuni Island in 2010 achieved 92% coverage [11]; 11 community clusters were randomly selected by multi-stage sampling as vaccinated comparators and surveyed in September 2013. Group 3, Convalescent cases: Sequential recently blood-culture confirmed typhoid cases in the Central Division were identified from national surveillance and hospital records and approached to seek informed consent for blood sampling. Further blood-culture confirmed cases diagnosed previously were identified during visits to the cases’ villages or residences and invited to participate after validation with national surveillance records. Between November 2013 and April 2014 up to three convalescent blood samples were collected at minimum one-month intervals.

Headmen, health workers and other community leaders were visited to seek agreement to participate and arrange visiting with the full study team. No communities declined participation. A team of experienced, multilingual Fijian field workers was trained and questionnaires piloted. Interviews were conducted in iTaukei, English or Hindi at the preference of the interviewee. Trained phlebotomists collected venous blood samples.

For group 1, cluster numbers per administrative Division were proportional to the resident typhoid non-immunised population. Contiguous nursing zones serving approximately 1,000 to 10,000 people were selected with probability proportional to population size [51] by random number generation using Ministry of Health administrative records. Community clusters were randomly selected within selected nursing zones from unweighted lists, in the absence of detailed population data.

Households were randomly selected within each community and an occupant aged ≥1 year was randomly selected (to reduce correlation compared with recruiting multiple residents) using random number tables. Health registries were used for this sampling where available; Otherwise, in rural village-like clusters, Expanded Programme on Immunization-derived sampling of houses was conducted in randomly selected directions (by pen-spin) from community centre points [52]. For street clusters, random starting points and set sampling intervals were determined following rapid house enumeration.

Household occupancy was *de facto* previous night residency as per Fiji census methods. Study information was provided. All adult participants provided written informed consent. Parents/guardians provided written informed consent on behalf of all child participants (under the age of 18 years old). Written assent was also provided by children aged 12 years and above. Exclusion criteria were clotting disorders, medical anticoagulation or severe medical disorders that would preclude safe participation.

For group 1 residents in Viti Levu, age- stratified sampling (strata size proportional to national population) was used for representativeness across age groups after field data review identified potential age imbalances in some clusters on Vanua Levu and Taveuni. If the selected participant was temporarily absent from a house e.g. for work or school, data collectors revisited later in the day after their expected time of return. If a whole household was absent, an alternative house was randomly selected from the health registry, or by geographical proximity.

### Sample size

A sample size of 1,600 was proposed for group 1, giving for 7% seroprevalence confidence intervals (CI) for age band groups of 200, if seroprevalence was 40%, at alpha = 0.05. If age bands had design effect of two due to non-independence within clusters, CI would be sufficiently precise at 10%. Expected seroprevalence levels were informed by prior work on Taveuni (Eric Nilles, data on file).

### Laboratory methods

Enzyme-linked immunosorbent assays (ELISAs) to detect *S*. Typhi Vi-polysaccharide antigen-specific IgG in human serum samples were performed as described previously [53] (provided by Sclavo Behring Vaccines Institute for Global Health, Siena, Italy). Briefly, ELISA plates were coated overnight with 1μg/ml of Vi polysaccharide antigen. Coated plates were washed and blocked with 5% fat-free milk solution. Following blocking, plates were washed and incubated with serum diluted at 1:200 at room temperature (RT) for 2 hours. Plates were washed and incubated with secondary antibody, alkaline phosphatase-conjugated anti-human IgG at RT for one hour. Finally, p-Nitrophenyl phosphate (SigmaFAST N1891, Sigma-Aldrich, United Kingdom) substrate was added for 30 minutes at RT and absorbance was read at dual wavelengths (405 nm and 490 nm) using an automated microplate reader (Biorad). Optical densities (OD) from blank control wells were subtracted from all sample absorbance values prior to estimation of serum titers from a standard curve. We selected 96 random Fijian plasma samples and subjected them to the anti-Vi ELISA. Twenty samples of these samples (with an OD >2.5) were pooled (standard plasma) and used to generate a standard curve. One ELISA Unit (EU) was defined as the reciprocal of the standard dilution (made by 10 2-fold dilutions of the standard plasma) that gave an absorbance value equal to 1 in this assay. All samples were tested at the Oxford University Clinical Research Unit in Ho Chi Minh City, Vietnam.

### Data analysis

A surveillance seropositivity threshold was determined using a mixed effects model of serial titres in the convalescent cases group. Models were fitted by maximum likelihood estimation (ML), using a random-intercept fixed-slope above a threshold value and random intercept time-invariant model below, signifying antibody returning to a baseline level. Data from two patients with titres at the upper limit of detection (25,000 EU) were excluded leaving 70 titres from 28 patients. Thresholds were compared using Akaike’s information criterion (AIC).

Data were entered in EpiData 3.1 [54] and analysed in R 3.3 [55]. Seroprevalences were calculated using intra-cluster correlation coefficients (ICCs) and design effects determined on log titres with clustering at the primary sample unit. Putative risk factors for seropositivity were estimated with Huber-White robust standard errors, clustered on the same, using the “rms” package[56]. A multivariable model was developed from univariable risk factors with *p*-values of less than 0.25, after-regrouping sparse cells for numerical stability, using a backward stepwise approach fitted by AIC, with deletion of observations with missing data. Potential collinearity was assessed by linear-adjusted generalized variance inflation factors (GVIF) in the “CAR” package [57,58], and variables were removed if GVIF^(1/(2*Df)) was over 2 and not considered epidemiologically important to retain. Data were considered insufficient to examine possible risk factors associated with titres that may indicate typhoid carriage. For comparison to age-based seroprevalence, typhoid fever cumulative incidence expected across the life-course was estimated with binomial confidence intervals from confirmed cases notified in 2008–2014 to the national surveillance system at the Fiji Centre for Communicable Disease Control, Mataika House, Suva.

## Results

**Fig 1:**
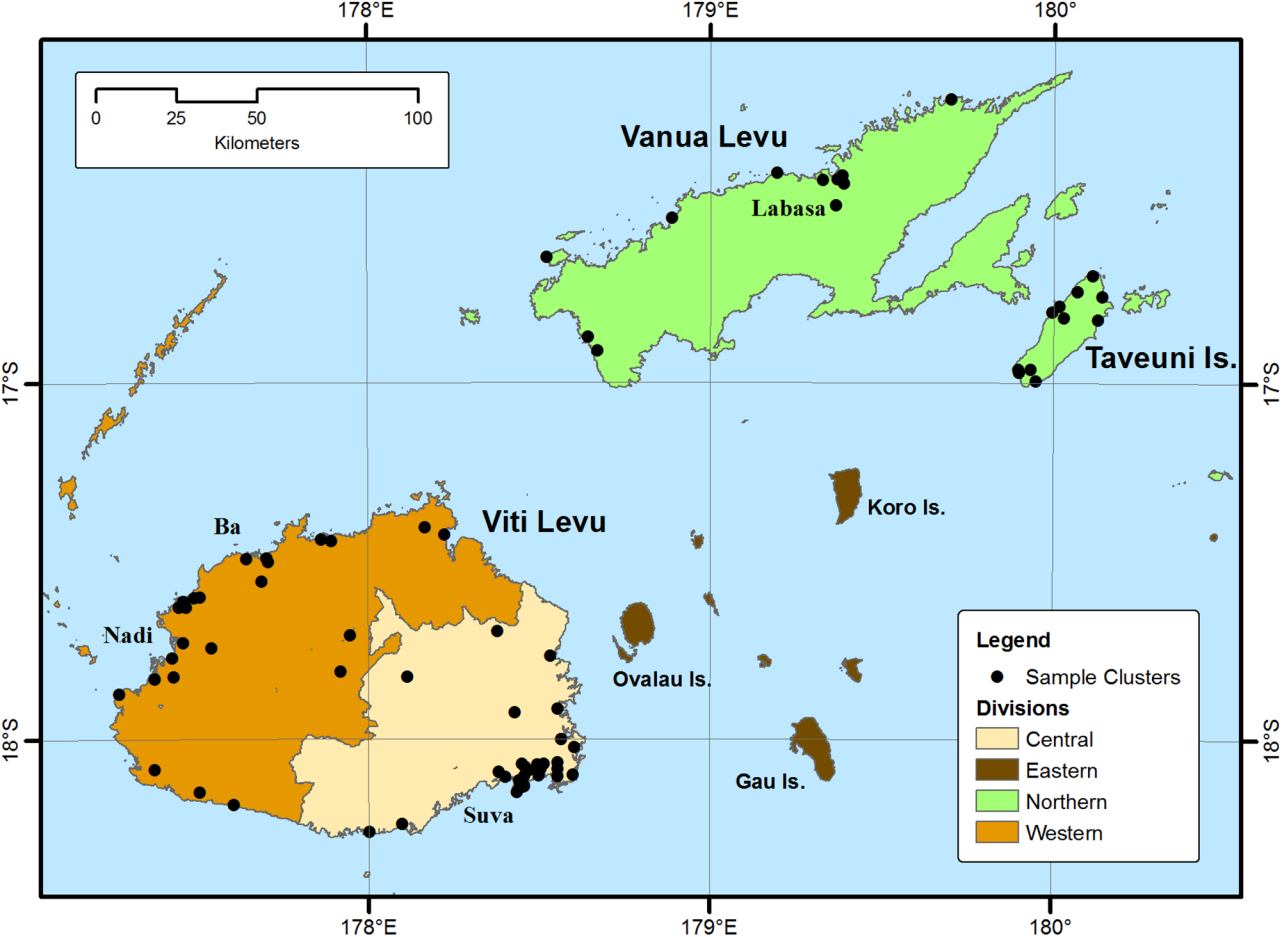
Administrative Divisions and Cluster sites on mainland Fiji (Viti Levu and Vanua Levu) and Taveuni islands.

Group 1: Sixty-four mainland clusters in unvaccinated areas of Viti Levu and Vanua Levu were visited for this sero-survey (Fig 1). Of 1,565 people approached, five declined and 1,560 were enrolled. There were no exclusions on medical grounds. A serum IgG titre against Vi polysaccharide (anti-Vi IgG) could not be attained for 29 participants (median age 23, IQR 6 to 34; 19/29 female; 25/29 iTaukei) but was determined in 1,531 individuals (98%; age range 1 to 85 years, median 29, IQR 16 to 48; 820/1,530 (54%) female; 1,164/1,530 76%) iTaukei; see Table 1; non-responses excluded).

**Table 1:** Group 1. Demographics of mainland Viti Levu and Vanua Levu (unvaccinated areas) survey participants.

Variable	Value
Number of participants assayed	1531
Age (median, IQR)	29 (16–48)
1 to 14	343 (22·4%)
15–34	554 (36·2%)
35–54	384 (25·1%)
55+	250 (22·9%)
Female	820 (53·6%)
Clusters	64
Central Division	28
Northern Division	11
Western Division	25
Participants per cluster (SD)	23·9 (2·5)
iTaukei	1164 (76·1%)
Indo-Fijian	338 (22·1%)
Other	28 (1·8%)
House income <100 FJD/wk	548 (36.3%)
100–199	490 (32.5%)
200–299	296 (19.6%)
300–399	61 (4.0%)
400+	81 (5.4%)
Self-report previous vaccination against typhoid	103 (6.7%)
Self-report previous typhoid fever	20 (1.3%)

For Group 2, on Taveuni Island, the location for a vaccination campaign, 277 people were approached and 256 participants (127 (49.6%) female) in 11 clusters enrolled, with nil excluded. All provided samples that were successfully assayed for anti-Vi IgG (Table 2 and Fig 1).

**Table 2:** Group2. Demographics of Taveuni island (Vi-polysaccharide vaccinated area) survey participants.

Variable	Value
Number of participants assayed	256
Age (median, IQR)	36 (24 to 52)
1 to 14	32 (12.5%)
15–34	90 (35.3%)
35–54	85 (33.3%)
55+	48 (18.8%)
Female	127 (50%)
Clusters	11
Participants per cluster (SD)	23.3 (3.3)
iTaukei	220 (86.3%)
Indo-Fijian	27 (10.6%)
Other	8 (3.1%)
House income <100 FJD/wk	91 (36.5%)
100–199	99 (39.8%)
200–299	22 (8.8%)
300–399	12 (4.8%)
400+	14 (5.6%)
Self-report previous vaccination against typhoid	54 (21.1%)
Self-report previous typhoid fever	5 (2.0%)

Group 3: Thirty-seven patients with recent blood-culture confirmed typhoid provided one or more samples that were assayed for anti-Vi IgG (19 (51.4%) female, median age 30, IQR 14 to 42) (Table 3); 30 provided two or more blood samples; and 19 provided three samples. Median duration from reported fever onset to first sample collection was 187 days (IQR 132 to 272 days).

**Table 3:** Group3. Demographics of convalescent confirmed typhoid cases.

Variable	Value
Number of participants assayed	37
Age (median, IQR)	30, 14 to 42
5 to 14	10 (27.0%)
15–34	12 (32.4%)
35–54	12 (32.4%)
55–74	3 (8.1%)
Female	19 (51.4%)
iTaukei	36 (97.3%)
Indo-Fijian	0
Other (Pacific Islander)	1 (2.7%)

Mixed model seropositivity threshold estimation in Group 3 (convalescent typhoid cases) exhibited best fit at 64 EU (S1 Fig and S1 Table). The ICC and design effect per Group 1 mainland cluster were 0.09 and 3.03, respectively. Across the five-year age bands (S2 Table), the Group 1 mean ICC and design effect were 0.13 and 1.09, respectively.

At the 64 EU threshold, 32.3% of Group 1 mainland participants (95%CI 28.2 to 36.3%) were seropositive for anti-Vi IgG (S3 Table), compared to 71.5% (95% CI 62.1% to 80.9%) of Group 2 (Taveuni island). For sensitivity analysis, at a threshold of 100 EU, 17·7% of the Group 1 mainland participants (95%CI 14·4 to 21.0%) were seropositive, and 58.6% (95% CI 48.4% to 68.8%) of Group 2 (Taveuni island) (S3 Table). To determine a rough estimate of carriage prevalence within Group 1 (mainland), we examined those with the highest anti-Vi IgG titres; 2.8% (1·4 to 4·2%) of the sampled population had an antibody titre of 500 EU or above and 1.4% (0.4% to 2·4%) of the sampled population had an antibody titre of 1,000 EU or above (S3 Table).

**Fig 2:**
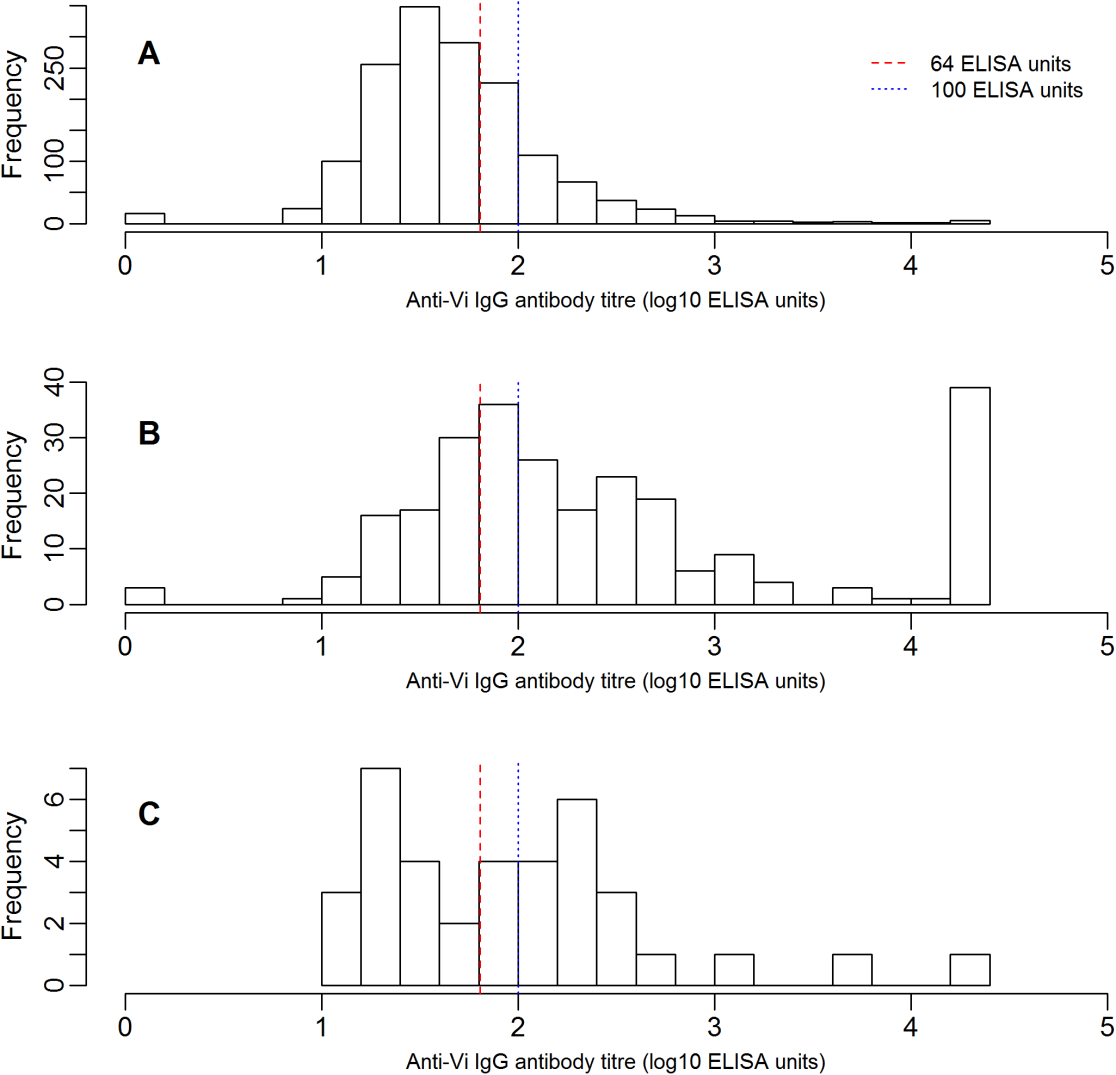
Distributions of log10 anti-Vi IgG antibody titres in A) Group 1: residents of Fiji mainland Viti Levu and Vanua Levu islands; B) Group 2: residents of Taveuni island, where a vaccination campaign with Vi-polysaccharide injection was conducted three years previously; and C) Group 3: recovering cases of culture-confirmed typhoid. Red vertical line denotes 64 ELISA unit seropositivity threshold determined from case antibody kinetic analysis; blue line denotes 100 ELISA unit threshold used in sensitivity analysis. Case titres are mean log titre if multiple samples collected, range 68 to 645 days from fever onset.

The anti-Vi IgG titre distributions are shown in Fig 2 for each surveyed group. The distribution of antibody titres in group 2 (Taveuni island) was shifted rightward relative to the Group 1 (mainland) titres, as would be predicted with the mass administration of Vi-PS vaccine on Taveuni. Thirty-nine (15%, 11.2 to 20.4%) of the 256 Group 2 (Taveuni) participants had Vi IgG titres at the upper limits of detection. In group 3, the convalescent typhoid cases, titres were bimodal, with the higher peak above the modal titre for the mainland group.

**Fig 3:**
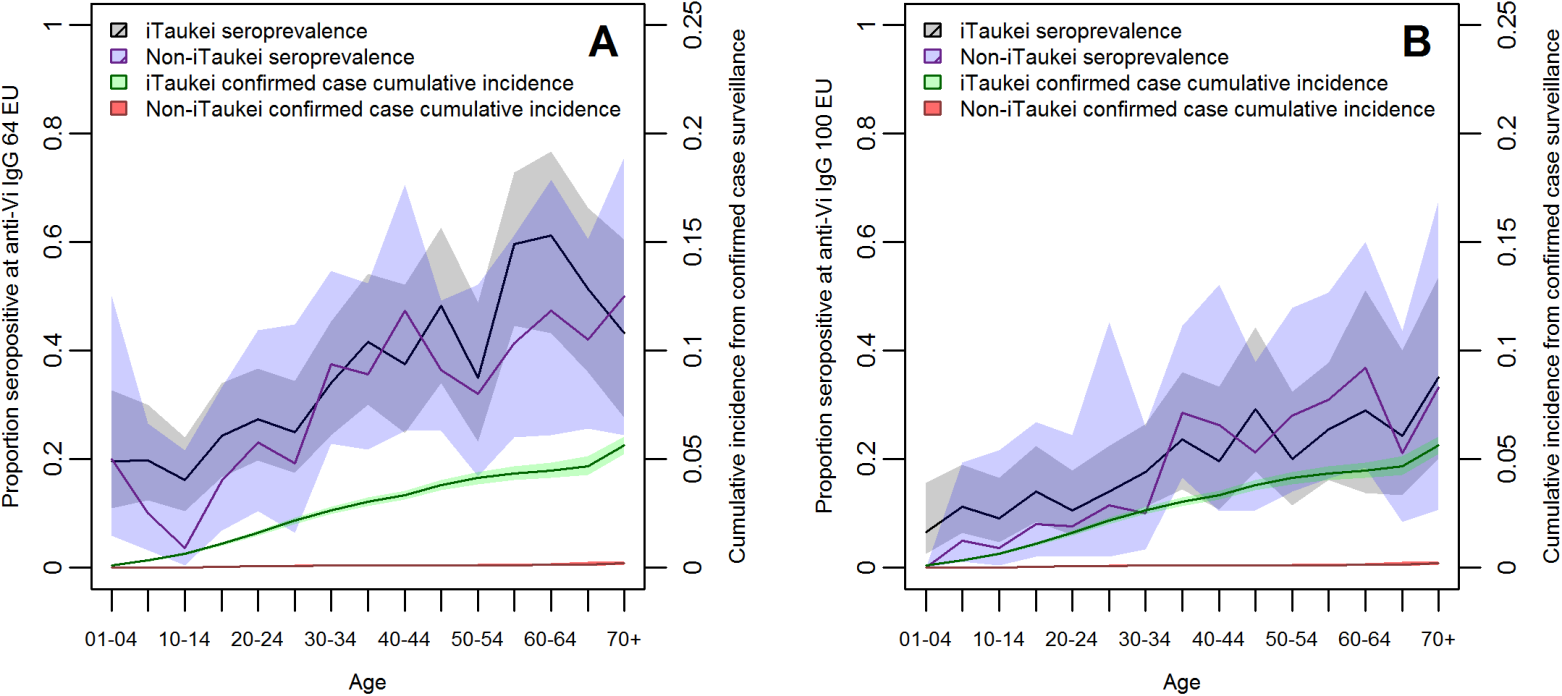
Seroprevalence of anti-Vi IgG by age and ethnicity (iTaukei and non-iTaukei) at A) 64 ELISA units (case-fitted threshold) and B) 100 ELISA units (sensitivity analysis). Each panel also indicates confirmed case cumulative incidence by ethnicity. Shared areas denote 95% confidence intervals.

Age trends for unvaccinated iTaukei and non-iTaukei ethnic groups each showed increasing seroprevalence with age at 64 EU threshold (group 1 mainland; Fig 3). These increased from approximately 20% in the youngest age bands to 50% in the oldest. In sensitivity analysis at 100 EU threshold, age and ethnicity trends were comparable, with seroprevalence rising from <10% in younger groups to approximately 30% in the oldest age brackets. Notably, for each ethnic group, seroprevalence by age band was substantially higher than the equivalent cumulative incidence that would arise if considering only confirmed cases, more than ten-fold in iTaukei Fijians and several hundred-fold in non-iTaukei Fijians.

Multivariable analysis of group 1 (mainland) found several factors were significantly associated with seropositivity at a 64 EU anti-Vi IgG threshold after adjusting for potential confounders (Table 4). Western Division residents had an adjusted odds ratio (OR) of 0.6 (95%CI 0.4 to 0.8) for seropositivity in comparison to the Central Division. Age association with seropositivity was retained, with an adjusted OR of 1.3 (95% CI 1.2 to 1.4) for every ten-year increase. Individuals with pit sewage systems had an adjusted OR of 1.6 (95% 1.1 to 2.3, *p* = 0.01) for seropositivity in comparison to participants with septic tanks. Residence in a settlement rather than residential housing had an adjusted OR 1.6 (95% CI 1.0 to 2.7) for seropositivity.

**Table 4:** Risk factors by adjusted odds ratios for anti-Vi IgG seropositivity at 64 ELISA units for mainland Viti Levu and Vanua Levu by cluster-robust multivariable logistic regression.

Variable	Value	OR	95% CI	p-value	
Division or island	Central Division	Baseline			
	Western Division	0.58	0.41 to 0.83	0.0027	**
	Vanua Levu	0.74	0.46 to 1.17	0.19	
Age	Per decade	1.31	1.23 to 1.40	<0.0001	***
Ethnicity	Other vs iTaukei	0.79	0.54 to 1.14	0.21	
Community type	Residential				
	Village	1.07	0.61 to 1.89	0.82	
	Settlement	1.63	1.00 to 2.65	0.048	*
Rurality	Urban	baseline			
	Peri-urban	0.65	0.41 to 1.01	0.055	
	Rural	1.17	0.72 to 1.88	0.53	
Home sewage	Septic tank	Baseline			
	Piped sewer	1.07	0.77 to 1.48	0.69	
	Pit	1.62	1.12 to 2.32	0.01	*
	Elsewhere	0.82	0.39 to 1.72	0.60	
Typhoid vaccination self-report	Yes	1.34	0.91 to 1.96	0.14	
Typhoid diagnosed, self-report	Yes	1.66	0.77 to 3.50	0.18	

*p<0.05

**p<0.01

***p<0.001

n = 1436 complete records

After adjustment, no significant association with seropositivity was observed at p<0.05 for ethnicity, community type, rural residence, self-reported typhoid vaccination, or self-reported diagnosis of typhoid fever. “Home toilet type” was excluded from consideration for multivariable analysis: pour-flush (water seal) toilets were found to be associated with seropositivity on univariable analysis, however these are installed in response to disease outbreaks and so are confounded by indication (Fiji National Taskforce on Control of Outbreak-Prone Diseases, personal communication 2015). Other candidate risk factors identified on univariable screening (S4 Table) were not retained in the final model, including sex, drinking water sources, kava consumption, bathing or washing in rivers and typhoid cases within the household or social network.

## Discussion

This seroepidemiological survey found seroprevalence of IgG against the Vi antigen of *S*. Typhi of 32.3% (95%CI 28.2 to 36.3%) in mainland Fiji: one to two orders of magnitude higher than would be predicted from case notifications. Seroprevalence increased with age, suggesting established endemic transmission. Both iTaukei and non-iTaukei ethnic groups exhibit similar seroprevalences across age groups, in contrast to notified disease, disproportionately reported from iTaukei Fijians. Some very high titres suggest that carriage occurs. The higher anti-Vi seroprevalence from Vi-PS vaccinated settings also informs the use of anti-Vi IgG as a surveillance marker in unvaccinated populations.

Our population-representative survey design strengthens external validity of seroprevalence estimates over convenience sampling, particularly for age-based inference, as children are rarely blood donors or inpatients. A limitation is the use of a single antigen due to resource availability, mitigated by mixed model determination of a surveillance titre threshold.

The proportion non-iTaukei (24%, specifically Indo-Fijian (22%)), was lower than expected from census data [13]. Potentially a greater proportion of Indo-Fijians reside in larger communities within nursing zones than documented in the sampling frame, or in vaccinated areas; higher emigration and lower fertility rates may also contribute [13]. Post-stratification weighting was not considered appropriate given demographic trend uncertainties since the 2007 census and sparse sub-nursing zone population records. Representativeness was addressed through survey design, and clustering through design-effects (which were modest) and cluster-robust regression. The slight excess of females in the survey may be due to different residency patterns, such as male overnight residency in agricultural shelters.

Vi IgG titres can be compared across studies, despite incomplete international assay standardisation [59]. The Fiji results contrast to two Vi ELISA serosurveys from Kathmandu, Nepal. The first found rising serum bactericidal activity with age, suggesting a similar acquisition of exposure with age, but found no age trend in anti-Vi IgG. [60] The second, using an assay similar to that applied in Fiji, reported high anti-Vi IgG in all age groups, suggesting hyperendemicity [61]. In Cape Town, South Africa, where typhoid was considered endemic, 40% of sampled unvaccinated 9 year olds were found to have anti-Vi IgG titres believed to be protective [62]. In contrast, we found mean seroprevalence in Fijian 5 to 9 and 10 to 14 year olds was not more than 20% (Fig 3) suggesting a lower force of infection, if antibody thresholds are comparable. This would also be consistent with lower confirmed case incidence [12,62].

Incomplete seroconversion (as seen in other settings[63]) and waning anti-Vi IgG titres observed amongst convalescent cases in our study suggest that multiple infections, symptomatic or otherwise, may be required for the establishment of sustained immunity to typhoid fever and the corresponding seroprevalence patterns observed in the Fijian mainland group. This concurs with prevailing conceptualisation[64,65], and experimental study [66] as well as recent models used to estimate vaccine impact [21,67] and elucidate transmission determinants in Malawi [23]. Papua New Guinea saw a similar upturn in typhoid notifications in the 1980-90s, also from a low-level, sporadic baseline, [68] with rise in population O antigen also observed [69], suggesting an overall increase in transmission; longitudinal seroepidemiology may likewise be informative in Fiji.

Ingestion of a large dose of *S*. Typhi can overwhelm naturally-acquired (and vaccine-derived) immunity [64,66,70,71], and so age- and ethnically-differential exposure to high and low dose inocula is one mechanism by which divergence between serological and confirmed-case data may be explained, if for example, iTaukei adolescents and young adults ingest larger inocula through exposure to particular foods. Such patterns might also be compatible with genetic differences in typhoid susceptibility, potentially mediated by HLA-type [72], with reduced susceptibility in Fijians of Indian descent, whose South-Asian ancestors may have experienced many millennia longer exposure to *S*. Typhi than iTaukei Fijians’ forebears [73–75].

Our multivariable analysis suggest that settlement residency and pit latrines use may be risk factors for *S*. Typhi infection. Whilst infrastructure upgrades may have multiple public health benefits, typhoid prevention should be planned with consideration for findings emerging from case-control and environmental health research [50,76]. Widespread subclinical infection, both transient and chronic, as suggested by this serosurvey, suggests that whilst systematic public health management of cases and outbreaks and early diagnosis and treatment of patients remain of vital importance to reduce morbidity and mortality from typhoid fever in Fiji, a focus on these alone may be insufficient to eliminate transmission. Alongside continued socio-economic development and expanded access to infrastructure for sanitation, water supplies and handwashing with soap, programmatic vaccination may be amongst interventions necessary to bring about effective typhoid control in Fiji.

## Supporting information

S1 Fig Serial anti-Vi IgG titres from convalescent confirmed typhoid cases. (TIFF)

S1 Table AIC by maximum likelihood for fixed effect model thresholds for anti-Vi IgG antibody waning to the threshold level in culture-confirmed typhoid cases. (DOCX)

S2 Table Design effects and ICC by 5-year age band, mainland Viti Levu and Vanua Levu. (DOCX)

S3 Table Seroprevalence for anti-Vi IgG at different ELISA unit thresholds amongst mainland residents (by self-reported vaccine status), Taveuni island residents and convalescent cases. (DOCX)

S4 Table Univariable risk factors for seropositivity with anti-Vi IgG on mainland Viti Levu & Vanua Levu at 64 ELISA unit threshold. (DOCX)

S1 File Supplementary information. (PDF)

S2 File STROBE checklist. (DOC)
