# Supplementary figures and images for: A cross-sectional seroepidemiological survey of typhoid fever in Fiji
Source: PLoS Negl Trop Dis. 2017 Jul 20;11(7):e0005786. doi: 10.1371/journal.pntd.0005786 (PMC5549756; doi:10.1371/journal.pntd.0005786)

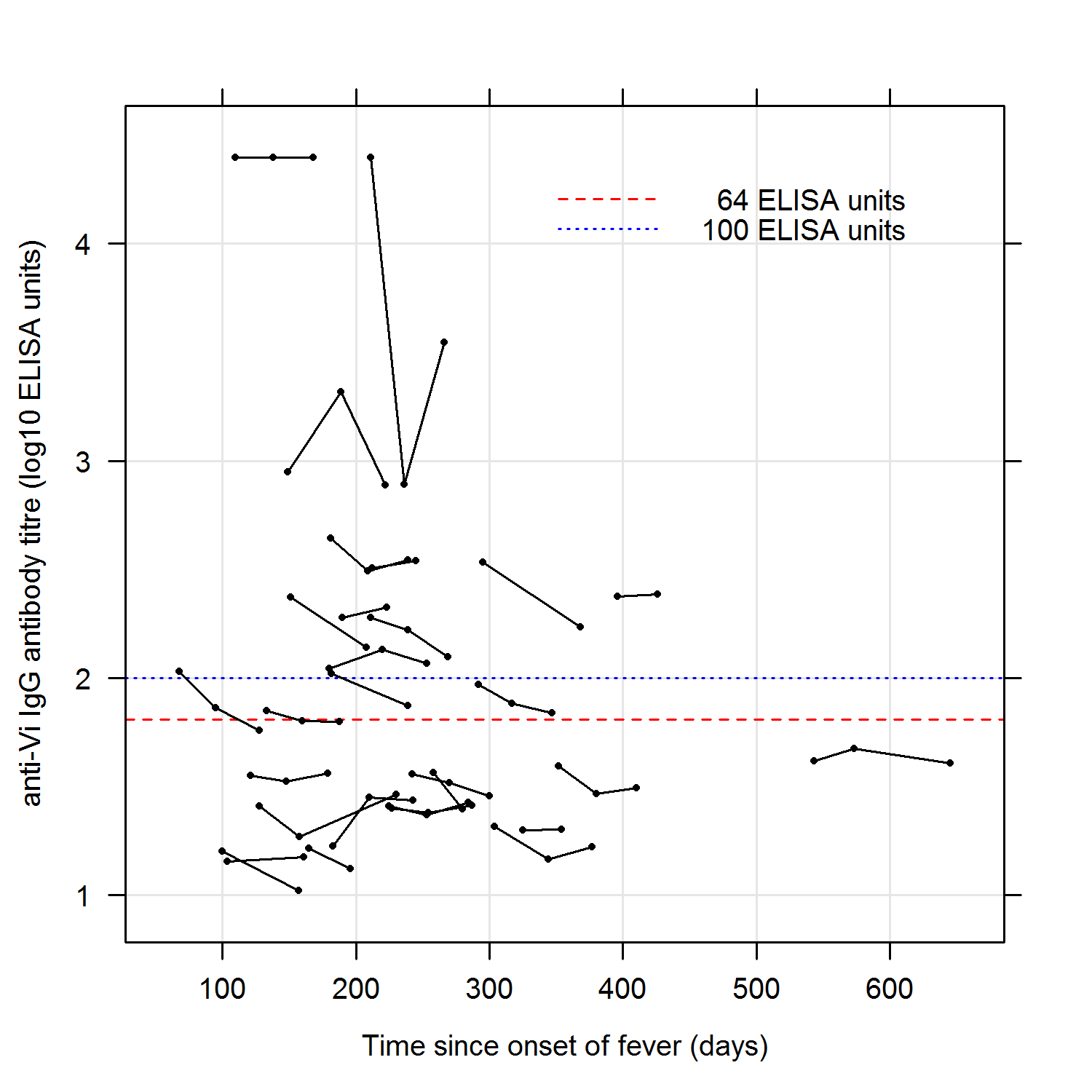

Supplement: S1 Fig — (TIFF) [file pntd.0005786.s001.tiff]
