# Supplementary material for: A cross-sectional seroepidemiological survey of typhoid fever in Fiji
Source: PLoS Negl Trop Dis. 2017 Jul 20;11(7):e0005786. doi: 10.1371/journal.pntd.0005786 (PMC5549756; doi:10.1371/journal.pntd.0005786)
Supplement: S1 Table — (DOCX) [file pntd.0005786.s002.docx]

| **Titre threshold (ELISA units)** | **AIC** | **Δ AIC from best fit** |
| --- | --- | --- |
| 16 | -24.48 | *29.95* |
| 32 | -46.29 | *8.13* |
| 64 | -54.43 | *0.00* |
| 100 | -45.07 | *9.36* |
| 150 | -44.59 | *9.84* |
| 200 | -34.22 | *20.20* |
| 250 | -21.07 | *33.36* |
