# Supplementary material for: A cross-sectional seroepidemiological survey of typhoid fever in Fiji
Source: PLoS Negl Trop Dis. 2017 Jul 20;11(7):e0005786. doi: 10.1371/journal.pntd.0005786 (PMC5549756; doi:10.1371/journal.pntd.0005786)
Supplement: S2 Table — (DOCX) [file pntd.0005786.s003.docx]

| Age band | Design effect | ICC |
| --- | --- | --- |
| 01-04 | 1.07 | 0.30 |
| 05-09 | 1.15 | 0.14 |
| 10-14 | 1.11 | 0.10 |
| 15-19 | 1.09 | 0.08 |
| 20-24 | 1.06 | 0.05 |
| 25-29 | 1.06 | 0.06 |
| 30-34 | 1.40 | 0.39 |
| 35-39 | 1.08 | 0.11 |
| 40-44 | 1.07 | 0.24 |
| 45-49 | 1.24 | 0.46 |
| 50-54 | 1.06 | 0.13 |
| 55-59 | 0.97 | -0.10 |
| 60-64 | 1.00 | -0.02 |
| 65-69 | 1.03 | -0.30 |
| 70+ | 0.94 | 0.45 |
