# Supplementary material for: A cross-sectional seroepidemiological survey of typhoid fever in Fiji
Source: PLoS Negl Trop Dis. 2017 Jul 20;11(7):e0005786. doi: 10.1371/journal.pntd.0005786 (PMC5549756; doi:10.1371/journal.pntd.0005786)
Supplement: S3 Table — (DOCX) [file pntd.0005786.s004.docx]

| **Group** | **Number (proportion, 95% confidence interval)**  **(by design effect for mainland and Taveuni)** |
| --- | --- |
| All mainland survey participants | 1531 |
| Titre ≥ 64 | 494 (32.3%, 28.2 to 36.3%) |
| Titre ≥ 100 | 271 (17.7%, 14.4 to 21.0%) |
| Titre ≥ 500 | 43 (2.8%, 1.4 to 4.2%) |
| Titre ≥ 1000 | 21 (1.4%, 0.4 to 2.4%) |
|  |  |
| Mainland unvaccinated (self-report) | 1304 (excludes don’t know) |
| Titre ≥ 64 | 410 (31.4%, 27.0 to 35.8%) |
| Titre ≥ 100 | 223 (17.1%, 13.5 to 20.7%) |
| Titre ≥ 500 | 37 (2.8%, 1.4 to 4.4%) |
| Titre ≥ 1000 | 18 (1.4%, 0.3 to 2.5%) |
|  |  |
| Mainland vaccinated (self-report) | 103 |
| Titre ≥ 64 | 42 (40.8%, 31.0 to 50.5%) |
| Titre ≥ 100 | 25 (24.3%, 15.7 to 32.8%) |
| Titre ≥ 500 | 5 (4.9%, 0.6 to 9.1%) |
| Titre ≥ 1000 | 3 (2.9%, 0 to 6.3%) |
|  |  |
| Taveuni island | 256 |
| Titre ≥ 64 | 183 (71.5%, 62.1 to 80.9%) |
| Titre ≥ 100 | 150 (58.6%, 48.4 to 68.8%) |
| Titre ≥ 500 | 72 (28.1%, 18.8 to 37.5%) |
| Titre ≥ 1000 | 57 (22.3%, 13.6 to 30.9%) |
|  |  |
| Convalescent typhoid cases | 37 |
| Mean titre ≥ 64 | 21 (56.8%, 39.6% to 72.5%) |
| Mean titre ≥ 100 | 17 (45.9%, 29.8% to 62.9%) |
| Mean titre ≥ 500 | 4 (10.8%, 3.5% to 26.3%) |
| Mean titre ≥ 1000 | 3 (8.1%, 2.1% to 23.0%) |
|  |  |
