# Supplementary material for: A cross-sectional seroepidemiological survey of typhoid fever in Fiji
Source: PLoS Negl Trop Dis. 2017 Jul 20;11(7):e0005786. doi: 10.1371/journal.pntd.0005786 (PMC5549756; doi:10.1371/journal.pntd.0005786)
Supplement: S4 Table — (DOCX) [file pntd.0005786.s005.docx]

| **Variable** | **Value** | **Count** | **OR** | **95% CI** | **P-value** |  |
| --- | --- | --- | --- | --- | --- | --- |
| Division or island | Central Division | 667 | Baseline |  |  |  |
|  | Western Division | 607 | 0.68 | 0.48 to 0.97 | 0.035 | * |
|  | Vanua Levu | 257 | 1.24 | 0.85 to 1.81 | 0.26 |  |
| Ethnicity | iTaukei | 1164 | Baseline |  |  |  |
|  | Other | 366 | 0.91 | 0.64 to 1.29 | 0.58 |  |
| Age | Per decade | 1530 | 1.28 | 1.21 to 1.36 | <0.0001 | *** |
| Sex | Male | 710 | Baseline |  |  |  |
|  | Female | 820 | 0.95 | 0.75 to 1.20 | 0.67 |  |
| Household size | Unit increase | 1499 | 0.93 | 0.88 to 0.98 | 0.0078 | ** |
| Community type | Residential | 430 | Baseline |  |  |  |
|  | Village | 656 | 1.24 | 0.86 to 1.79 | 0.26 |  |
|  | Settlement | 444 | 1.41 | 0.99 to 2.02 | 0.06 | ^ |
| Rurality | Urban | 500 | Baseline |  |  |  |
|  | Periurban | 262 | 0.62 | 0.44 to 0.88 | 0.0067 | ** |
|  | Rural | 763 | 1.30 | 0.90 to 1.88 | 0.16 | ^ |
| Income  (FJD household^-1^ week^-1^) | 0-99 | 548 | baseline |  |  |  |
|  | 100-199 | 490 | 0.98 | 0.31 to 1.64 | 0.91 |  |
|  | 200-299 | 296 | 0.96 | 0.72 to 1.35 | 0.77 |  |
|  | 300-399 | 61 | 0.78 | 0.48 to 1.27 | 0.32 |  |
|  | 400+ | 81 | 0.91 | 0.49 to 1.67 | 0.75 |  |
| Drink tap water at home | 4+d/wk | 1427 | baseline |  |  |  |
|  | Never | 87 | 1.68 | 1.15 to 2.47 | 0.0075 | ** |
|  | Monthly | 3 | 1.09 | 0.10 to 12.17 | 0.95 |  |
|  | 1-3d/wk | 8 | 1.3 | 0.32 to 5.33 | 0.71 |  |
| Drink river water | Never | 1451 | Baseline |  |  |  |
|  | < monthly | 12 | 0.72 | 0.24 to 2.13 | 0.55 |  |
|  | Monthly | 11 | 1.23 | 0.45 to 3.31 | 0.69 |  |
|  | 1-3d/wk | 9 | 2.68 | 0.73 to 9.93 | 0.14 | ^ |
|  | 4+d/wk | 36 | 1.92 | 1.04 to 3.55 | 0.037 | * |
| Drink kava | Any vs never | 608:913 | 1.43 | 1.12 to 1.84 | 0.0048 | ** |
|  | At least monthly vs less than monthly | 1018:503 | 1.28 | 1.02 to 1.61 | 0.037 | * |
| Kava shared with how many people at last consumption? | Not applicable | 895 | Baseline |  |  |  |
|  | Zero to nine | 387 | 1.28 | (1.02 to 1.62) | 0.0367 | * |
|  | Ten or more | 235 | 1.79 | (1.24 to 2.58) | 0.0018 | ** |
| Bath or swim in rivers | Never | 1065 | Baseline |  |  |  |
|  | < monthly | 187 | 0.89 | 0.59 to 1.34 | 0.57 |  |
|  | Monthly | 99 | 0.82 | 0.52 to 1.29 | 0.39 |  |
|  | 1-3d/wk | 83 | 0.90 | 0.51 to 1.56 | 0.70 |  |
|  | 4+/wk | 92 | 1.40 | 0.81 to 2.41 | 0.23 | ^ |
| Home toilet | Flush | 1174 | Baseline |  |  |  |
|  | Water seal (pour flush) | 244 | 1.52 | 1.03 to 2.25 | 0.035 | * |
|  | Pit or bucket | 106 | 1.38 | 0.98 to 1.93 | 0.62 |  |
| Sewage | Piped sewer | 285 | Baseline |  |  |  |
|  | Septic tank | 991 | 1.21 | 0.90 1.62 | 0.20 | ^ |
|  | Pit | 138 | 2.07 | 1.31 3.26 | 0.0019 | * |
|  | Elsewhere | 42 | 1.17 | 0.62 2.22 | 0.63 |  |
| Toilet location | Indoor | 913 | Baseline |  |  |  |
|  | Detached | 610 | 1.22 | 0.94 to 1.58 | 0.13 | ^ |
| Shared toilet | Private | 1379 | Baseline |  |  |  |
|  | Shared | 106 | 0.86 | 0.59 to 1.26 | 0.44 |  |
| Soap available after household toilet use | No | 133 | baseline |  |  |  |
|  | Yes, reported | 1199 | 0.84 | 0.52 to 1.35 | 0.47 |  |
|  | Yes, seen | 189 | 0.78 | 0.45 to 1.35 | 0.38 |  |
|  | Yes, seen/reported | 1388 | 0.83 | 0.52 to 1.33 | 0.44 |  |
| Self-reported soap use | No | 414 | Baseline |  |  |  |
|  | Yes | 1091 | 1.00 | 0.77 to 1.29 | 0.99 |  |
| Household tap | No | 102 | Baseline |  |  |  |
|  | Yes | 1422 | 0.72 | 0.46 to 1.12 | 0.15 | ^ |
| Typhoid vaccination self-report | No + Don’t know | 1304+112 | Baseline |  |  |  |
|  | Yes | 103 | 1.51 | 1.07 to 2.12 | 0.019 | * |
| Typhoid diagnosis self-report | No | 1453 | Baseline |  |  |  |
|  | Yes | 20 | 2.15 | 0.96 to 4.82 | 0.062 | ^ |
| Typhoid in the household, self-report | No + Don’t know | 1447+6 | Baseline |  |  |  |
|  | Yes | 20 | 0.63 | 0.19 to 2.12 | 0.46 |  |
| Know at least one person who has had typhoid | No + Don’t know | 1341+36 |  |  |  |  |
|  | Yes | 93 | 1.51 | 1.01 to 2.25 | 0.042 | * |

^p<0.25, * p<0.05, **p<0.01, ***p<0.001

n=1531
