# Supplementary material for: A cross-sectional seroepidemiological survey of typhoid fever in Fiji
Source: PLoS Negl Trop Dis. 2017 Jul 20;11(7):e0005786. doi: 10.1371/journal.pntd.0005786 (PMC5549756; doi:10.1371/journal.pntd.0005786)
Supplement: S1 File — (PDF) [file pntd.0005786.s006.pdf]

Supplementary information

Self-reported typhoid immunisation history was considered to be non-informative, as only 21% of Taveuni islanders reported vaccination against an expected coverage of over 90%.(1) Furthermore, 100 (6.7%) mainland residents reported receiving a typhoid vaccine, in locations where coverage was predicted to be zero with only five of these participants having possibly previously resided in areas covered by the 2010 vaccination campaign. A subgroup sensitivity analysis was conducted in the 1,428 mainland participants who reported no history of typhoid immunisation. Seropositivity for anti-Vi IgG in this group was observed at thresholds of 1:64, 1:100, 1:500 and 1:1,000 for 31.4% (27.0 to 35.8%), 17.7% (13.5% to 20.7%), 2.8% (1.4 to 4.4%) and 1.4% (0.3 to 2.5%) respectively, indicating no difference from the full mainland survey group.

References

1. Scobie HM, Nilles E, Kama M, Kool JL, Mintz E, Wannemuehler KA, et al. Impact of a Targeted Typhoid Vaccination Campaign Following Cyclone Tomas, Republic of Fiji, 2010. Am J Trop Med Hyg. 2014 Apr 7;90(6):1031–8.
