# Supplementary material for: A cross-sectional seroepidemiological survey of typhoid fever in Fiji
Source: PLoS Negl Trop Dis. 2017 Jul 20;11(7):e0005786. doi: 10.1371/journal.pntd.0005786 (PMC5549756; doi:10.1371/journal.pntd.0005786)
Supplement: S2 File — (DOC) [file pntd.0005786.s007.doc]

STROBE Statement—checklist of items that should be included in reports of observational studies

|  | Item No | Recommendation | Where | What |  |  |
| --- | --- | --- | --- | --- | --- | --- |
| **Title and abstract** | 1 | (*a*) Indicate the study’s design with a commonly used term in the title or the abstract | Title | “Cross-sectional… survey” |  |  |
|  |  | (*b*) Provide in the abstract an informative and balanced summary of what was done and what was found | Abstract | “To characterize the epidemiology of typhoid exposure in Fiji, we conducted a cross-sectional sero-epidemiological survey measuring IgG against the Vi antigen of *S*. Typhi to estimate the effect of age, ethnicity, and other variables on seroprevalence. Epidemiologically relevant cut-off titres were established using a mixed model analysis of data from recovering culture-confirmed typhoid cases. A total of 1,787 participants were enrolled and their plasma assayed for anti-Vi IgG; 1,531 of these were resident in mainland areas that had not been previously vaccinated against *S*. Typhi (seropositivity 32.3% (95%CI 28.2 to 36.3%)), 256 were resident on Taveuni island, which had been previously vaccinated (seropositivity 71.5% (95%CI 62.1 to 80.9%)). The seroprevalence on the Fijian mainland is one to two orders of magnitude higher than expected from confirmed case surveillance incidence, suggesting substantial subclinical or otherwise unreported typhoid. We found no significant differences in seropositivity prevalences by ethnicity, which is in contrast to disease surveillance data in which the indigenous iTaukei Fijian population are disproportionately affected. Using multivariable logistic regression, seropositivity was associated with increased age” |  |  |
| Introduction | | |  |  |  |  |
| Background/rationale | 2 | Explain the scientific background and rationale for the investigation being reported | pp4-6 | “[A]n expert meeting was convened in 2012 by the Fijian Ministry of Health, with support from Australian Aid, to “develop, prioritise and implement a comprehensive control and prevention strategy”.[11] Analysis of knowledge gaps identified that a serological survey across multiple demographic groups could inform vaccination policy.[11]  Seroepidemiological surveys can be used to determine population immunity, pathogen exposure and disease susceptibility, as well as determining disease and exposure related risk factors.[23] Conducted alongside clinical and/or laboratory surveillance, seroepidemiology can help quantify surveillance under- or over-ascertainment, including for enteric diseases.[24–27] Setting-specific immunity and carriage are important in determining typhoid transmission dynamics;[28–30] however, the seroepidemiological methods to attain this are underexploited.[24] This may be in part due to concerns about the sensitivity and specificity of serology for typhoid, which historically has not demonstrated sufficient discriminatory power for individual-level clinical diagnosis, [31] (though recent methods may offer promise [32]) as well as concerns about the specificity of assays for carriage detection [33–35] and the existence of multiple immunological pathways to immunity against typhoid fever.[36] Seroepidemiological surveys utilising assays based on purified, pharmaceutical-grade Vi polysaccharide, the virulence antigen expressed by S. Typhi, for detection of anti-Vi IgG antibody may offer a more reliable approach by avoiding cross-reactivity that arises when Vi antigen preparations contain other bacterial antigens.[37,38] Furthermore, high anti-Vi titres likely indicate chronic carriage in response to prolonged immune stimulus. [37,39–41]” |  |  |
| Objectives | 3 | State specific objectives, including any prespecified hypotheses | p6 | “To characterize and better define the immunoepidemiology of typhoid infection in Fiji, with the aim of informing effective and efficient control measures”. |  |  |
| Methods | | |  |  |  |  |
| Study design | 4 | Present key elements of study design early in the paper | p6 | “a representative, multi-stage, clustered, cross-sectional seroepidemiological survey of the Fijian mainland for S. Typhi, including demographic data and data on potential risk factors for typhoid fever” |  |  |
| Setting | 5 | Describe the setting, locations, and relevant dates, including periods of recruitment, exposure, follow-up, and data collection | pp7-8 | Group 1, Mainland: Sixty-four communities were randomly selected by multi-stage sampling on the two most populous Fijian islands, Viti Levu and Vanua Levu. These were visited for serological sampling and questionnaire interviews from September to December 2013. Vaccinated populations on these two islands were excluded to avoid confounding of interpretation of serological responses to natural exposure. Eastern Division, with a population of under 40,000 spread across multiple small islands,[56] was not visited for logistical reasons.  Group 2, Taveuni: A vaccination campaign had been conducted on Taveuni Island in 2010; 11 communities from this location were randomly selected by multi-stage sampling as vaccinated comparators and surveyed in September 2013.  Group 3, Convalescent cases: Sequential recently confirmed typhoid cases in the Central Division were identified in October 2013 from national surveillance and hospital records and approached to seek informed consent for blood sampling. Further confirmed cases diagnosed previously were identified during visits by the field team to the convalescent cases’ villages or residences and invited to participate after validation with national surveillance records. Between November 2013 to April 2014 up to three blood samples were collected from cases at a minimum of one-month intervals. |  |  |
| Participants | 6 | (*a*) *Cohort study*—Give the eligibility criteria, and the sources and methods of selection of participants. Describe methods of follow-up  *Case-control study*—Give the eligibility criteria, and the sources and methods of case ascertainment and control selection. Give the rationale for the choice of cases and controls  *Cross-sectional study*—Give the eligibility criteria, and the sources and methods of selection of participants |  | multi-stage cluster-sample survey…cluster numbers per geographical Division (Northern, Central, Western) were proportional to the resident typhoid non-immunised population… nursing zones were selected with probability proportional to population size… random number generation in Microsoft Excel 2007, using Ministry of Health administrative records. Communities were randomly selected from within the nursing zones from unweighted lists…  single occupant aged one year or older per household was randomly invited to join the study, with selection using random number tables…  Registries held by nurses or community health workers were used for household and participant sampling where available; when not available, geographical sampling was performed. In rural village-like clusters, EPI-derived sampling of houses was conducted in randomly selected directions (by pen-spin) from the community’s central point.[58] For clusters on streets, random sides of roads, starting points, and directions of progress were selected following a rapid appraisal of house numbers to enable selection of all households with equal probabilities.  Household occupancy was *de facto*, based on residency in the household the previous night |  |  |
|  |  | (*b*) *Cohort study*—For matched studies, give matching criteria and number of exposed and unexposed  *Case-control study*—For matched studies, give matching criteria and the number of controls per case |  | NA |  |  |
| Variables | 7 | Clearly define all outcomes, exposures, predictors, potential confounders, and effect modifiers. Give diagnostic criteria, if applicable | S4 Table, pp14-17, Table 4. | “anti-Vi IgG titre”… “Threshold estimation using a mixed model of sero-reversion amongst the recovering typhoid cases exhibited best fit at 64 ELISA units”  Univariable:  Division or island  Ethnicity  Age  Sex  Household size  Community type  Rurality  Income (FJD household-1 week-1)  Drink tap water at home  Drink river water  Drink kava  Kava shared with how many people at last consumption?  Bath or swim in rivers  Home toilet  Sewage  Toilet location  Shared toilet  Soap available after household toilet use  Self-reported soap use  Household tap  Typhoid vaccination, self-report  Typhoid diagnosis self-report  Typhoid in the household, self-report  Know at least one person who has had typhoid  Multivariable exposure factors and confounders:  Division or island  Age  Ethnicity  Community type  Rurality  Home sewage  Typhoid vaccination self-report  Typhoid diagnosed, self report  “Home toilet type” was excluded from consideration for multivariable analysis: pour-flush (water seal) toilets were found to be associated with seropositivity on univariable analysis, however these are installed in response to disease outbreaks and so are confounded by indication (Fiji National Taskforce on Control of Outbreak-Prone Diseases, personal communication 2015). Other candidate risk factors identified on univariable screening (S4 Table) were not retained in the final model, including sex, drinking water sources, kava consumption, bathing or washing in rivers and typhoid cases within the household or social network. |  |  |
| Data sources/ measurement | 8* | For each variable of interest, give sources of data and details of methods of assessment (measurement). Describe comparability of assessment methods if there is more than one group | pp7-9, S4 Table, Table 4. | *“*questionnaire interviews… A team of experienced, multilingual Fijian field workers was trained and questionnaires piloted prior to the survey. Interviews were conducted in iTaukei, English or Hindi at the preference of the interviewee. Venous blood samples were collected by trained phlebotomists.”  “Enzyme-linked immunosorbent assays (ELISAs) to detect *S.* Typhi Vi-polysaccharide antigen-specific IgG in human serum samples were performed as described previously[60] (provided by Sclavo Behring Vaccines Institute for Global Health, Siena, Italy) with the exception of blocking with 5% non-fat milk (instead of BSA). Briefly, ELISA plates were coated overnight with 1μg/ml of Vi polysaccharide antigen. Coated plates were washed and blocked with 5% fat-free milk solution. Following blocking, plates were washed and incubated with serum diluted at 1:200 at room temperature (RT) for 2 hours. Plates were then washed and incubated with the secondary antibody, alkaline phosphatase-conjugated anti-human IgG at RT for one hour. Finally, p-Nitrophenyl phosphate (SigmaFAST N1891, Sigma-Aldrich, United Kingdom) substrate was added for 30 minutes at RT and absorbance was read at dual wavelengths (405 nm and 490 nm) using an automated microplate reader (Biorad). Optical densities (OD) from blank control wells were subtracted from all sample absorbance values prior to estimation of serum titers from a standard curve. |  |  |
| Bias | 9 | Describe any efforts to address potential sources of bias | pp7-8 | Selection bias:  multi-stage cluster-sample survey…cluster numbers per geographical Division (Northern, Central, Western) were proportional to the resident typhoid non-immunised population… nursing zones were selected with probability proportional to population size… random number generation in Microsoft Excel 2007, using Ministry of Health administrative records. Communities were randomly selected from within the nursing zones from unweighted lists…  single occupant aged one year or older per household was randomly invited to join the study, with selection using random number tables…  Registries held by nurses or community health workers were used for household and participant sampling where available; when not available, geographical sampling was performed. In rural village-like clusters, EPI-derived sampling of houses was conducted in randomly selected directions (by pen-spin) from the community’s central point.[58] For clusters on streets, random sides of roads, starting points, and directions of progress were selected following a rapid appraisal of house numbers to enable selection of all households with equal probabilities.  Household occupancy was *de facto*, based on residency in the household the previous night.”  Confounding bias:  Seroprevalences were calculated using design effects determined on log titres with clustering at the primary sample unit. Putative risk factors for seropositivity were estimated with Huber-White robust standard errors, clustered on the same, using the “rms” package.[63] A multivariable model was developed from univariable risk factors with *p*-values of less than 0.25, after-regrouping sparse cells for numerical stability, using a backward stepwise approach fitted by AIC, with deletion of observations with missing data. Potential collinearity was assessed by linear-adjusted generalized variance inflation factors in the “CAR” package,[64,65] and variables were removed if GVIF^(1/(2*Df)) was over 2 and not considered epidemiologically important to retain. |  |  |
| Study size | 10 | Explain how the study size was arrived at | p9 | “A study sample size of 1,600 was proposed for the mainland group, as this would allow for 7% confidence intervals for seroprevalence for age band groups of 200, if seroprevalence was 40%, at alpha = 0.05. If non-independence within age bands within clusters gave rise to a design effect of two, then the confidence intervals would be 10%, which was deemed sufficient precision.” |  |  |
| Quantitative variables | 11 | Explain how quantitative variables were handled in the analyses. If applicable, describe which groupings were chosen and why | Table 4. | “logistic regression” as per dichotomised seroprevalence y-variable.  In the tables it apparent from the x-variable values that these are all handled as factors, which the exception of age which is numerical. Age has been reported as decades in line with Greenland and Pearce etc to avoid reporting tiny annualised odds ratios with very strong p-values. |  |  |
| Statistical methods | 12 | (*a*) Describe all statistical methods, including those used to control for confounding | pp7-8 | Seroprevalences were calculated using design effects determined on log titres with clustering at the primary sample unit. Putative risk factors for seropositivity were estimated with Huber-White robust standard errors, clustered on the same, using the “rms” package.[63] A multivariable model was developed from univariable risk factors with *p*-values of less than 0.25, after-regrouping sparse cells for numerical stability, using a backward stepwise approach fitted by AIC, with deletion of observations with missing data. Potential collinearity was assessed by linear-adjusted generalized variance inflation factors in the “CAR” package,[64,65] and variables were removed if GVIF^(1/(2*Df)) was over 2 and not considered epidemiologically important to retain. |  |  |
|  |  | (*b*) Describe any methods used to examine subgroups and interactions | pp7-8 | As above |  |  |
|  |  | (*c*) Explain how missing data were addressed | p8 | “deletion of observations with missing data” |  |  |
|  |  | (*d*) *Cohort study*—If applicable, explain how loss to follow-up was addressed  *Case-control study*—If applicable, explain how matching of cases and controls was addressed  *Cross-sectional study*—If applicable, describe analytical methods taking account of sampling strategy | pp8,18 | “Seroprevalences were calculated using design effects determined on log titres with clustering at the primary sample unit. Putative risk factors for seropositivity were estimated with Huber-White robust standard errors, clustered on the same…Post-stratification weighting was not considered appropriate given uncertainties in demographic changes since the 2007 census and sparse population records within nursing zones which could result in inappropriate adjustment. Representativeness was addressed through the design of the survey, and clustering through use of design-effects (which were modest) and cluster-robust regression.” |  |  |
|  |  | (*e*) Describe any sensitivity analyses | Fig 3, Fig2 | “Seroprevalence of anti-Vi IgG by age and ethnicity (iTaukei and non-iTaukei) at A) 64 ELISA units (case-fitted threshold) and B) 100 ELISA units (sensitivity analysis)”  Fig 2 denotes fitted 64 EU threshold and 100 EU sensitivity threshold. |  |  |

Continued on next page

| Results | | | Where | What |
| --- | --- | --- | --- | --- |
| Participants | 13* | (a) Report numbers of individuals at each stage of study—eg numbers potentially eligible, examined for eligibility, confirmed eligible, included in the study, completing follow-up, and analysed | p12 | “1,560 people enrolled, a serum IgG titre against Vi polysaccharide (anti-Vi IgG) was determined in 1,531 individuals (98%). |
|  |  | (b) Give reasons for non-participation at each stage | p12 | “a serum IgG titre against Vi polysaccharide (anti-Vi IgG) was determined in” |
|  |  | (c) Consider use of a flow diagram | NA | Considered and ruled out. |
| Descriptive data | 14* | (a) Give characteristics of study participants (eg demographic, clinical, social) and information on exposures and potential confounders | Tables 1-3, S4 Table | See tables |
|  |  | (b) Indicate number of participants with missing data for each variable of interest | p12, Table 4, S4 Table | “Of 1,560 people enrolled, a serum IgG titre against Vi polysaccharide (anti-Vi IgG) was determined in 1,531 individuals (98%)”. Number of complete analysable cases listed in T4 as 1436. Missingness in univariable table S4 can be calculated by deducting figures presented from the 1531 given in the text. |
|  |  | (c) *Cohort study*—Summarise follow-up time (eg, average and total amount) | pp12-14, S1 Fig | Regarding patients studied:  Page 12: “median duration from reported fever onset to first sample collection was 187 days (IQR 132 to 272 days)”  Figure 2 “Case titres are mean log titre if multiple samples collected, range 68 to 645 days from fever onset.”  Full data presented in S2 Fig |
| Outcome data | 15* | *Cohort study*—Report numbers of outcome events or summary measures over time |  | *N/A* |
|  |  | *Case-control study—*Report numbers in each exposure category, or summary measures of exposure |  | *N/A* |
|  |  | *Cross-sectional study—*Report numbers of outcome events or summary measures | *14, S3 Table* | *“*Using the defined seropositivity cut-off (64 EU threshold), 32.3% of mainland participants (95%CI 28.2 to 36.3%) were seropositive for anti-Vi IgG (S3 Table),” |
| Main results | 16 | (*a*) Give unadjusted estimates and, if applicable, confounder-adjusted estimates and their precision (eg, 95% confidence interval). Make clear which confounders were adjusted for and why they were included | S4 Table, Table 4, p10 | See tables in full.  Variable retention was by “using a backward stepwise approach fitted by AIC, with deletion of observations with missing data. Potential collinearity was assessed by linear-adjusted generalized variance inflation factors in the “CAR” package,[64,65] and variables were removed if GVIF^(1/(2*Df)) was over 2 and not considered epidemiologically important to retain.” |
|  |  | (*b*) Report category boundaries when continuous variables were categorized | Fig 3.  S4 Fig. | Age boundaries reported.  Frequency boundaries reported. |
|  |  | (*c*) If relevant, consider translating estimates of relative risk into absolute risk for a meaningful time period | Fig 3. | Cumulative incidence reported alongside seroprevalence.  Table 4/S4: Not appropriate to convert to absolute risks. |
| Other analyses | 17 | Report other analyses done—eg analyses of subgroups and interactions, and sensitivity analyses | pp14-15, Fig 2, 3, S3 Table | Seropositivity threshold of 100 EU shown.  “As a sensitivity analysis, when a higher anti-Vi IgG threshold of 100 EU was used, 17·7% of the mainland participants (95%CI 14·4 to 21.0%) were seropositive, compared to 58.6% (95% CI 48.4% to 68.8%) on Taveuni (S3 Table).”…  “Age and ethnicity trends were comparable when a sensitivity analysis at a higher threshold (100 EU) was performed with seroprevalence rising from <10% in younger groups to approximately 30% in the oldest age brackets. At both thresholds there was some suggestion that iTaukei seroprevalence may be higher than non-iTaukei seroprevalence, though the differences were not statistically significant at the 0.05 level. Notably, for both ethnic groups, the seroprevalence by age band was substantially higher than the equivalent cumulative incidence that would arise if considering only confirmed cases, more than ten-fold in iTaukei Fijians and several hundred-fold in non-iTaukei Fijians.” |
| Discussion | | |  |  |
| Key results | 18 | Summarise key results with reference to study objectives | p18 | “This seroepidemiological survey of the Fijian mainland, established in response to a rise in confirmed typhoid case notifications, found seroprevalence of IgG against the Vi antigen of S. Typhi of 32.3% (95%CI 28.2 to 36.3%): one to two orders of magnitude higher than would be predicted from case notifications. Seroprevalence increased with age, suggesting established endemic transmission. Both iTaukei and non-iTaukei ethnic groups exhibit similar seroprevalences across age groups, with a possible slight excess amongst iTaukei, in contrast to disease which is disproportionately reported from iTaukei Fijians. A small number of very high titres suggests that carriage occurs. Multivariable logistic regression found seropositivity was associated with pit latrines compared with other sewage systems, and living in a settlement compared with residential housing or a village. Central Division and Vanua Levu island had higher seroprevalences than Western Division.” |
| Limitations | 19 | Discuss limitations of the study, taking into account sources of potential bias or imprecision. Discuss both direction and magnitude of any potential bias | Pp18-19 | “incomplete international standardisation of Vi assays”  “The proportion of the survey participants that was non-iTaukei (24%, specifically Indo-Fijian (22%)), was lower than expected from the last census.[66] This may be due to a greater proportion of Indo- Fijians residing in larger communities within nursing zones than documented in the sampling frame, due to migration from rural to urban area, or in areas that had been vaccinated; secular trends of a higher emigration rate and lower fertility rate for Indo-Fijians than iTaukei may also be have contributed.[56] Post-stratification weighting was not considered appropriate given uncertainties in demographic changes since the 2007 census and sparse population records within nursing zones which could result in inappropriate adjustment.” |
| Interpretation | 20 | Give a cautious overall interpretation of results considering objectives, limitations, multiplicity of analyses, results from similar studies, and other relevant evidence | Pp18-21 | Please see entire discussion. The priority is clearly given towards the age-based seroprevalence analysis, interpreted in conjunction with national case surveillance, consistent with objectives, rather than prioritising the cluster-robust regression. Similar data from Nepal and Kathmandu are discussed, and various permutations of the epidemiological implications for Fiji, including how follow-on and ongoing research will further reduce epidemiological unknowns. |
| Generalisability | 21 | Discuss the generalisability (external validity) of the study results | P18 | “Our investigation used a population-representative survey design rather than a convenience sample such as blood donors or hospital patients, or recent outbreak areas. This strengthens external validity of seroprevalence estimates, particularly for age-based inference, as children are rarely blood donors or inpatients.” |
| Other information | | |  |  |
| Funding | 22 | Give the source of funding and the role of the funders for the present study and, if applicable, for the original study on which the present article is based | P22 | Fieldwork was funded by the World Health Organization World Health Organization, Division of Pacific Technical Support and by the Chadwick Trust. CHW is supported by the UK Medical Research Council (grant MR/J003999/1). SB and RdA are funded by the Wellcome Trust of Great Britain. CLL was supported by a research grant from the Global Change Institute (607562) at The University of Queensland. Other than contributions by EJN, the funders had no role in the study design, analysis, interpretation or decision to publish. |

*Give information separately for cases and controls in case-control studies and, if applicable, for exposed and unexposed groups in cohort and cross-sectional studies.

**Note:** An Explanation and Elaboration article discusses each checklist item and gives methodological background and published examples of transparent reporting. The STROBE checklist is best used in conjunction with this article (freely available on the Web sites of PLoS Medicine at http://www.plosmedicine.org/, Annals of Internal Medicine at http://www.annals.org/, and Epidemiology at http://www.epidem.com/). Information on the STROBE Initiative is available at www.strobe-statement.org.
